# Contrast enhancement pattern predicts poor survival for patients with non-WNT/SHH medulloblastoma tumours

**DOI:** 10.1007/s11060-015-1779-0

**Published:** 2015-04-11

**Authors:** Maria Łastowska, Elżbieta Jurkiewicz, Joanna Trubicka, Paweł Daszkiewicz, Monika Drogosiewicz, Katarzyna Malczyk, Wiesława Grajkowska, Ewa Matyja, Bożena Cukrowska, Maciej Pronicki, Marta Perek-Polnik, Danuta Perek, Bożenna Dembowska-Bagińska

**Affiliations:** Department of Pathology, The Children’s Memorial Health Institute, Av. Dzieci Polskich 20, 04-730 Warsaw, Poland; Department of Radiology, The Children’s Memorial Health Institute, Av. Dzieci Polskich 20, 04-730 Warsaw, Poland; Department of Medical Genetic, The Children’s Memorial Health Institute, Av. Dzieci Polskich 20, 04-730 Warsaw, Poland; Department of Neurosurgery, The Children’s Memorial Health Institute, Av. Dzieci Polskich 20, 04-730 Warsaw, Poland; Clinic of Oncology, The Children’s Memorial Health Institute, Av. Dzieci Polskich 20, 04-730 Warsaw, Poland; The Department of Experimental and Clinical Neuropathology, Mossakowski Medical Research Centre, Polish Academy of Sciences, ul. Pawińskiego 5, 02-109 Warsaw, Poland

**Keywords:** Medulloblastoma, Location, Contrast enhancement, Survival

## Abstract

Recent studies revealed the biological heterogeneity of medulloblastoma, with the existence of at least four groups which are associated with several clinical and morphological features. We investigated for further correlations between molecular types, location of tumours, their contrast enhancement pattern and survival of patients. Altogether 76 tumours were analyzed and molecular subtypes were identified by immunohistochemistry using representative antibodies, detection of chromosome 6 monosomy and *CTNNB1* mutation. The site of the tumour was assessed on diagnosis using Magnetic Resonance images and intra-operative surgical reports. In addition, the gadolinium enhancement pattern was also investigated in pre-treatment tumours. Cerebellar hemispheric location was associated with SHH tumours (p < 0.001), as opposed to midline location being typical for WNT and non-WNT/SHH tumours. Remarkably, for patients with non-WNT/SHH tumours, the extensive gadolinium enhancement pattern (present in >75 % of tumour volume) predicted worse OS and EFS than for those with none/weak or heterogeneous enhancement (>10–75 % of tumour volume), (both p < 0.001). Our analysis indicates that distribution of the medulloblastoma tumours location is related to the biological characteristics of tumour. Importantly, the enhancement pattern of the tumour may be a clinically useful prognostic marker for patients with non-WNT/SHH medulloblastomas.

**Electronic supplementary material:**

The online version of this article (doi:10.1007/s11060-015-1779-0) contains supplementary material, which is available to authorized users.

## Introduction

Medulloblastoma is the most common malignant paediatric brain tumour. Recently, molecular studies confirmed the biological heterogeneity of the disease with the existence of at least four transcriptional groups: Wingless (WNT), sonic hedgehog (SHH), Group 3 and Group 4 tumours [1–3]. Correlations between the transcriptional groups, histological types and clinical features have also been identified. For example, patients with WNT tumours have a favourable outcome and, by contrast, patients with Group 3 tumours have the worst prognosis [2, 4, 5].

The distinctive transcriptional patterns may result, at least in part, from the fact that tumours may arise from different cells of origin [6–8]. Therefore the findings, based on mouse models experiments, should corroborate with a distinct location of tumours in humans. Gibson et al. [8] found that WNT tumours had midline location and were attached to the dorsal brainstem as opposed to SHH tumours which were located within the cerebellar hemispheres. Teo et al. [9] also found all WNT tumours in midline location, but only about half of SHH tumours were hemispheric. By contrast, very recent findings by Perreault et al. [10] showed that the majority of WNT tumours were located in the cerebellopontine angle/cerebellar peduncle and indicated this location as predictive for the WNT Group. In addition, they examined several radiologic features among which minimal or no gadolinium enhancement was associated with Group 4 tumours.

Because it is difficult presently to distinguish Group 3 from Group 4 tumours without an application of multi-genes assays based on methylation or genes expression signatures [11], other features should be tested within non-WNT/SHH tumours for their potential clinical value. In this study therefore we investigated if tumour location and enhancement pattern may serve as clinically useful surrogate markers in medulloblastoma. Our analysis confirmed that hemispheric location is typical for SHH tumours and that none/weak enhancement was associated with Group 4 tumours.

More importantly, since both MRI and molecular analyses were performed on the largest cohort of uniformly treated medulloblastoma patients from a single institution published to date, we were able to show for the first time that non-WNT/SHH tumours can be sub-classified further into different prognostic groups according to the gadolinium enhancement pattern.

## Materials and methods

### Patients

**Fig. 1:**
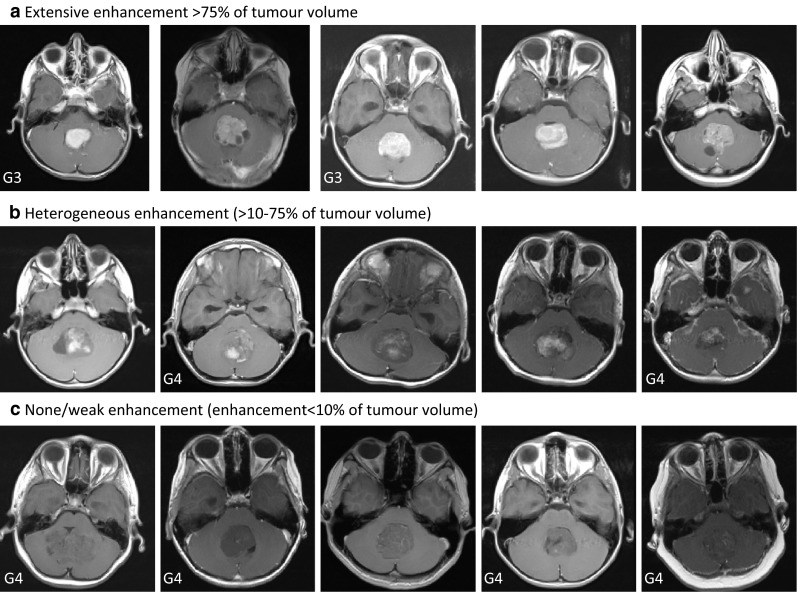
Representative transverse MR images showing gadolinium enhancement pattern in non-WNT/SHH medulloblastoma tumours. G3—molecular group 3, G4—molecular group 4

Seventy-six medulloblastoma patients treated in The Children’s Memorial Health Institute (CMHI) in Warsaw, Poland, according to the Polish Pediatric Neurooncology Group (PPNG) protocol (Fig. 1S) were included in the analysis. Informed consent was obtained to use tumour material according to the procedures outlined by the CMHI’s Ethical Committee. The sole criterion for patient inclusion was availability of both MR images and tumour tissue for analyses at diagnosis.

### Pathologic evaluation

Formalin-fixed paraffin embedded (FFPE) hematoxylin-eosin-stained slides were reanalysed by two neuropathologists (WG, EM) and paediatric pathologist (MP), according to the current WHO 2007 criteria [12]. Additional staining with reticulin was performed to distinguish truly nodular/desmoplastic (D/N) tumours from tumours with a presence of pseudonodules. Large cell/anaplastic tumours (LCA) were diagnosed where anaplastic features were identified in a majority of analysed areas. Antibodies against LIN28A (A177, #3978, Cell Signaling Technology) and SNF5 (Abcam #42503) were also applied in a subset of tumours with atypical location to verify medulloblastoma diagnosis.

### Molecular group detection

*Immunohistochemistry* was applied on FFPE tissue preparations according to established protocols at diagnosis using antibodies against β-catenin (DB #610154, 1:800) and GAB1 (Abcam #ab27439 and/or #59362, 1:100). Preparations were treated in a heat antigen retrieval citrate buffer for 20 min for both antibodies.

*Interface Fluorescence in situ hybridisation (FISH)* was performed on FFPE tissue preparations for detection of monosomy 6 using chromosome 6 Satelite Enumeration Probe (Kreatech) and *MYCC* amplification using Vysis MYC probe (Abbott, USA), according to the protocols of the manufacturer of the probes.

*Multiplex ligation*-*dependent probe amplification (MLPA)* was carried out on genomic DNA extracted from frozen tumour for detection of copy number changes of chromosome 6. The analysis was performed using the SALSA MLPA kit P301-A2 (MRC-Holland, Amsterdam, the Netherlands) according to the manufacturer’s protocol. Peak plots were visualized and normalized, and the dosage ratios were calculated using GeneMarker software v 2.2.0 (Soft Genetics, LLC, State Collage, PA, USA).

*Mutations in exon 3of CTNNB1* gene were detected in genomic DNA obtained from available frozen tumour tissues using the Sanger direct method. The PCR reactions were carried out with the following primers: CTNNB1_3F:CCCTGGCTATCATTCTGCTT and CTNNB1_3R:TCTCTTTTCTTCACCACAACATTT using Amplitaq Gold DNA Polymerase (Roche) under following conditions: 95 °C for 8 min; 35 cycles of 95 °C for 1 min; 57 °C for 5 min; 72 °C for 1 min then a final extension step of 72 °C for 7 min. Sequencing reactions were performed using a BigDye Terminator v.3.1 Cycle Sequencing Kit (Life Technologies) according to the manufacturer’s protocol. Sequencing products were analyzed in ABI Prism 3130 Genetic Analyzer (Applied Biosystems, Foster City, CA, USA). Sequences of the analyzed fragments were compared with the *CTNNB1* cDNA (GenBank RefSeq: NM_001904.3) using Mutation Surveyor software version 3.30 (Soft Genetics, LLC, State Collage, PA, USA). The positions of the identified nucleotide changes were determined based on comparison with the reference sequence, with the A of the ATG translation initiation codon designated as nucleotide +1.

Altogether, the transcriptional subtypes of tumours were identified as follows:

1. WNT tumours by presence of at least two features: positive nuclear reaction against β-catenin, chromosome 6 monosomy and/or *CTNNB1* mutation, as recommended by the International Medulloblastoma Working Group [11],
2. SHH tumours by presence of a positive reaction with anti-GAB1 antibody, as described by Ellison et al. [13],
3. Non-WNT/SHH tumours were the remaining tumours tested negative for the above features. This group included a subset of tumours previously analysed as part of a MAGIC cohort by an application of expression microarrays or NanoString technologies [4], and the results were exploited for Group 3 and Group 4 discrimination within our non-WNT/SHH tumours (Table 1S).

### Imaging analysis

Brain magnetic resonance imaging (MRI) studies were performed on a 1.5 T scanner (Sonata, Siemens) with a dedicated 8-channel head coil. The examination protocol included the following sequences and images:

- Transverse planes: TSE T2WI (3920/106), fl2d T1WI (234/4.76) [TR-repetition time/TE-echo time], and FLAIR (2200/8000/114) [IR-inversion time/TR/TE].
- Sagittal TSE T2WI (5450/139) and coronal TSE T2WI (5290/135) [TR/TE].
- Sagittal fl T1WI (237/4.76) and coronal fl T1WI (234/4.76) [TR/TE].

Matrix: 256 × 256 and 256 × 192, field of view: 160–256 mm, slice thickness: 3–5 mm, slice interval of 20–30 %.

Additionally, T1 spoiled gradient recalled images tfl3d_ns_IR_sag_iso were obtained before and after contrast medium administration: TI/TR/TE = 1100/1840/3.92 ms, voxel size: 1 ×1 × 1 mm, 192 slices, flip angle = 12. The contrast was injected in a standard dose 0.1 mmol/kg Gd-DTPA.

MR images were analysed at diagnosis by two experienced radiologists (EJ and KM). Tumour location was defined as midline (IVth ventricule, vermis), lateral in cerebellar hemispheres or cerebellopontine angle (CPA), and both lateral and middle location. Gadolinium enhancement pattern was assessed by visual inspection during the first sequence performed not later than 2 mins after contrast injection. It was defined as weak/none or present when more than 10 % of the tumour volume enhanced. Then, present enhancement was distinguished as extensive (>75 % of the tumour volume enhanced) or heterogeneous (>10–75 % of tumour volume enhanced).

### Review of neurosurgical reports

Operative reports from all 76 patients who underwent neurosurgery in the CMHI were reviewed by a neurosurgeon (PD). As with the MRI assessment, the location of the primary tumour was established as midline (IVth ventricule, vermis) or lateral (cerebellar hemispheres or CPA). In addition, extent of tumour resection (total, subtotal, partial), surgically suggested sites of tumour origin and sites of tumour invasion were taken into account.

### Statistical analysis

The Fisher Exact test was performed to establish associations between variables. Overall survival (OS) and event free survival (EFS) were calculated using Kaplan–Meier estimation and group comparisons were made using the log-rank test.

Univariate analyses within the non-WNT/SHH Group included presence of metastases at diagnosis according to Chang et al. [14], LCA pathology and extent of tumour resection. Backward step-wise Cox proportional hazards procedures were used to determine which variables have an independent effect on survival.

## Results

### Patients and tumours characteristics

The average age of 76 patients at diagnosis was 9 years, range 0.5–18 years. Forty-eight patients were males, 28 patients were females.

The pathological diagnosis of medulloblastoma was classic in 57 cases, LCA in 12 cases, DN in four cases, medulloblastoma tumour with extensive nodularity (MBEN) in two cases and in one case histopathological subtype was not determined.

Molecular groups included: six WNT tumours, nine SHH tumours and 61 non-WNT/SHH tumours. Among the latter group, seven tumours were identified as Group 3 and ten tumours as Group 4, according to previous MAGIC consortium investigation [4]. A summary of patients and tumour characteristics are presented in Tables 1 and 1S.

**Table 1:** Characteristics of 76 patients with medulloblastoma

	ALL	WNT	SHH	Non-WNT/SHH	*Non*-*WNT/SHH subset group 3*	*Non*-*WNT/SHH subset group 4*
No of patients	76	6	9	61	7	10
Age years						
Average (range)	9 0.5–18	8 5–12	6.1 0.5–18	9.5 0.5–18	7.1 5–11	11.4 7–16
0–3	10	0	6	4	0	0
>3–16	63	6	2	55	7	7
>16–18	3	0	1	2	0	3
Gender						
Male	48	2	6	40	5	6
Female	28	4	3	21	2	4
Histopathology						
Classic	57	6	2	49	6	9
LCA	12	0	1	11	1	1
DN (reticulin positive)	4	0	4	0	0	0
MBEN	2	0	2	0	0	0
na	1	–	–	1	–	–
Metastases						
M0M1	48	6	9	33	4	5
M2M3	26	0	0	26	2	5
na	2	–	–	2	1	–

*LCA* large cell/anaplastic, *DN* desmoplastic/nodular, *na* not available

### Location of tumour and molecular group

The location of the tumour was determined by both MRI and is presented in Table 2. Lateral hemispheric location was significantly associated with SHH tumours (p < 0.001 SHH vs. other tumours).

**Table 2:** Location of tumour according to both MRI and surgery reports

Group	All	WNT	SHH	Non-WNT/SHH	*Subset group 3*	*Subset group 4*
No of patients	76	6	9	61	7	10
Location						
MRI lateral		1	6	1	0	0
Surgical	Hemisphere	–	6*	–	–	–
	CPA	1	–	1	–	–
MRI midline		5	2	58	7	10
Surgical	Vermis/IVth	5	2	55	6	10
	IV th	–	–	3	1	–
MRI Lateral + middle		–	1	2	0	0
Surgical	Hemisphere	–	–	1	–	–
	CPA/IVth	–	–	1	–	–
	Vermis/IVth	–	1	–	–	–

* Hemispheric location is significantly associated with SHH tumours (p < 0.001, Fisher Exact Test)

*CPA* cerebellopontine angle, *IVth* IVth ventricule, *na* not available

Lateral PCA location was identified in one out of six WNT tumours and in two out of 61 non-WNT/SHH tumours. All 17 tumours from Group 3 and Group 4 had midline location.

In three cases, MRI location included both lateral and middle structures and the surgical report was helpful in pointing to hemispheric or PCA location as the tumour’s original site (Table 2).

Atypical cases within the non-WNT/SHH Group included one infant with LCA diagnosis who had unusually high tumour location surgically reported as arising from superior medullary velum. The tumour displayed a positive reactions with anti-LIN28 and SNF5 antibodies, typical for a subset of PNET tumours [15]. The patient died due to rapid local recurrence despite previous total resection of the tumour and chemotherapy. Only one patient had restricted PCA location and another both PCA and midline location. Both patients are alive and disease free.

None of the SHH tumours had recorded invasion of the brain stem floor, including two cases with MRI midline tumours where lower vermis and IVth ventricule were surgically reported as the tumour location. Both of these patients were infants with DN or MBEN tumour pathology.

### Gadolinium enhancement pattern, biological group and survival

Seventy-five tumours with known gadolinium enhancement pattern were divided according to none/weak versus present enhancement to establish if these two features are typical for any particular molecular type. None/weak enhancement was present in three out of six WNT tumours, one out of eight SHH tumours and 29 out of 60 non-WNT/SHH tumours. Although the results were not statistically significant a trend towards more frequent presence of enhancement in SHH tumours was noticed (p = 0.09).

Then we focused on non-WNT/SHH tumours to assess if none/weak enhancement is associated with other features within this group (Table 3) and found that only Group 4 tumours were associated with none/weak enhancement (p = 0.03).

**Table 3:** Pattern of gadolinium enhancement in non-WNT/SHH tumours relative to other features

Enhancement pattern	None/weak <10 %	Present		
		Extensive >75 %	Heterogeneous >10–75 %	Fisher exact test
No of patients	29	20	11	
Histopathology				
LCA	3	7	1	p = 0.027
Classic	25	13	10	
na	1	–	–	
Metastases				
M0M1	17	11	4	ns
M2M3	11	8	7	
na	1	1	–	
Surgical resection				
Total	8	5	2	ns
Subtotal/partial	21	14	8	
na	–	1	1	
Subset of non-WNT/SHH				
Group 3	0	6	0	p = 0.0009
Group 4	6	1	3	p = 0.03

None/weak enhancement is associated with Group 4 tumours and extensive enhancement with Group 3 tumours and LCA pathology

*LCA*—large cell/anaplastic pathology, *na*—not available, *ns*—not significant

Because the presence of enhancement includes various degrees of tumour volume, we subdivided this characteristic further into two subcategories: extensive enhancement (>75 % of tumour volume) and heterogeneous enhancement (>10–75 % of tumour volume) and examples are shown in Fig. 1. In our series extensive enhancement pattern was significantly associated with Group 3 tumours (p = 0.0009, Table 3).

Since Group 3 and 4 tumours were associated with different survival rates of patients in previous studies [2, 4, 5], we hypothesised that tumour enhancement pattern alone may serve as a useful prognostic marker in the biologically relevant non-WNT/SHH group of medulloblastoma.

First, we confirmed that our patients treated according to the uniform PPNG protocol and identified in the previous MAGIC cohort [4] as Group 3 tumours (n = 14) had significantly worse EFS than patients with Group 4 tumours (n = 21), (p = 0.041, log-rank test).

Then we focused analyses on 60 patients with non-WNT/SHH tumours. Eleven patients were excluded from survival analysis based on the following merits: four patients below 3 years of age were treated according to separate protocol without OUN irradiation, four patients died due to treatment complications, three patients had lateral location of the tumour and for one patient follow up data were not available. MBEN pathology was not present in non-WNT/SHH tumours. Frequency of both LCA pathology and presence of M2M3 metastases were not statistically different between the excluded and analyzed group.

Therefore we analysed finally 49 patients with non-WNT/SHH tumours as one cohort for their survival rate according to enhancement pattern. All patients were above 3 years of age and had midline location of the tumour.

**Fig. 2:**
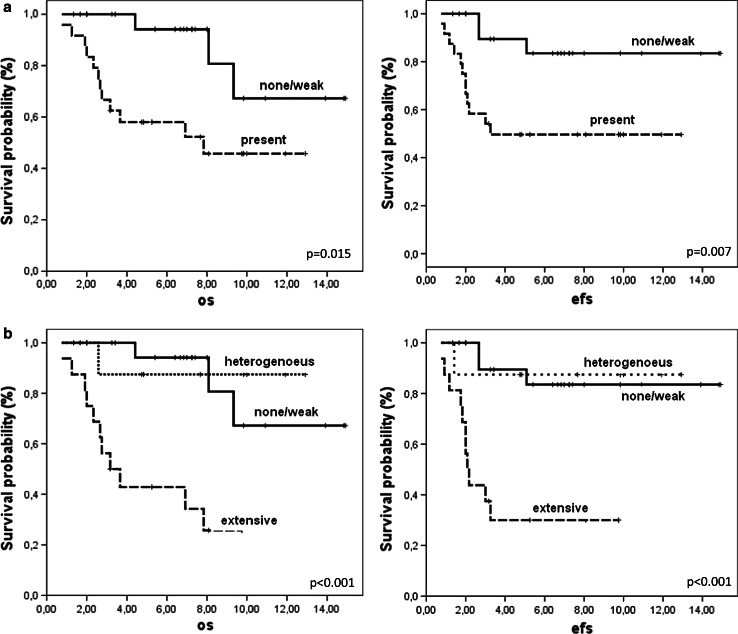
Survival of patients according to gadolinium enhancement pattern in non-WNT/SHH tumours. **a** OS and EFS according to none/weak (*continuous lines*) or present enhancement in >10 % of tumour volume (dashed lines) **b** OS and EFS according to none/weak (*continuous lines*), heterogeneous (>10–75 % of tumour volume, *dotted lines*) and extensive enhancement (>75 % of tumour volume, *dashed lines*). p values are presented for 5-years OS and EFS

Patients with none/weak enhancement had significantly better five-year OS and EFS than patients with the presence of enhancement (p = 0.015 and 0.007, respectively, Fig. 2a). Moreover, patients with extensive enhancement had even worse OS and EFS than those with none/weak or heterogeneous enhancement (both p < 0.001, Fig. 2b). Among extensively enhancing tumours, two had amplification of *MYCC* oncogene, what corroborates with a bad prognosis for those patients. After exclusion of the latter two cases from the analysis, extensive enhancement was still significantly associated with a worse survival rate (p < 0.001). Among other features, only the presence of LCA pathology was associated with worse five-year OS (28 vs. 53 % for classic pathology) and EFS survival (14 % vs. 78 % for classic pathology, both p < 0.001) in analyzed series, but not the presence of metastases (p = 0.28 for EFS) or extent of surgically assessed tumour resection (p = 0.23 for EFS). Backward step-wise Cox proportional hazards procedure indicated that extensive enhancement was slightly more significant than LCA pathology (p = 0.005 vs. p = 0.012 and HR = 5.8 vs. 4.1).

## Discussion

Our analysis confirmed that distribution of medulloblastoma location is not random but is related to the biological characteristics of the tumour. Summarising the previous three studies [8–10] and our results, it becomes clear that hemispheric cerebellar location can serve as surrogate marker for SHH medulloblastoma since it was not found in any of the 31 WNT tumours analysed to date and only in three out of the 157 non-WNT/SHH tumours analysed. It is likely that the latter three cases may display distinct biological characteristics and should be investigated further. However, SHH tumours are not only restricted to hemispheric location and, with the exception of the results of Gibson et al. [8], were also found in various numbers in the midline location, depending on the study cohort (up to 53 %). Our two patients with exclusively midline location were young children with DN or MBEN pathology what is consistent with the findings by Grammel et al. [7] that some SHH tumours may arise from the cochlear nuclei of the brainstem. However, none of them had surgically recorded invasion of the brain stem floor and the lower vermis was indicated as a site of the disease.

Our study also indicates that both midline and lateral sites can be found in WNT tumours, but only one out of six tumours had PCA location. Since WNT tumours are relatively rare, the proportion of different locations can differ depending on the study series, methods of group detection and patients’ demographics. Therefore, more cases need to be analysed to establish the original site for those tumours.

The largest group of non-WNT/SHH tumours was characterised by midline location where upper, lower or whole vermis was affected. Only few tumours had an atypical site in this group and additional tests should confirm medulloblastoma diagnosis in such cases.

In addition to tumour location, several MRI features were analysed in medulloblastoma so far [16–18], but they were not related to the molecular sub-grouping of disease discovered in recent years. The only study investigating such a relationship was published by Perreault et al. [10] on discovery cohort of 47 patients and found that Group 3 tumours had ill-defined tumour margins and Group 4 tumours had minimal or no enhancement. The latter feature was validated on post-contrast T1WI images in an additional 52 patients and served as a predictive marker for Group 3 and Group 4 identification. In our study, the smaller number of the total of 17 tumours from Group 3 and Group 4 still delivered similar results thus confirming the findings by Perreault et al. [10].

Furthermore, we also investigated if enhancement pattern may be associated with the survival of patients. Because there were only few patients with WNT and SHH tumours in our study due to the restricted availability of diagnostic MR images for analysis, we focused the investigation on non-WNT/SHH tumours only and found that patients with none/weak enhancement had significantly better OS and EFS than those with the presence of enhancement. Moreover, patients with extensive enhancement had even worse OS and EFS than those with none/weak or heterogeneous enhancement (p < 0.001).

The subdivision of gadolinium enhancement pattern was based deliberately on visual inspection only, without the use, for example, gradient-echo imaging analysis. After the exclusion of clearly visible cysts and cavities, it was possible to identify tumours with extensive enhancement in the majority of enhancing tumours. The threshold of 75 % sets up a clinically relevant feature and includes solid (> 90 % of tumour volume) enhancing tumours, as shown in the study by Perreault et al. [10] where, unfortunately, no relation to survival was presented. Heterogeneous enhancement, on the other hand, includes patchy patterns exposing possible areas of mineralization, hemorrhage or necrosis within tumours. All of this may be associated with distinctive biological characteristics of tumour or delivery of drugs what, in turn, perhaps have an impact on better treatment results for these patients. Nevertheless, heterogeneous tumours represented the smallest investigated group what require further collection of cases to confirm our findings.

It is not clear why extensive enhancement pattern correlates with poor survival of patients. Our analysis indicates that this pattern was associated with LCA pathology, molecular Group 3 and included two cases with *MYCC* amplification thus pointing to aggressive phenotype of tumours. Although recently recognised molecular Group 3 is associated with worse prognosis than Group 4, the significance of this observation depends on the study cohort [2, 4, 5]. Therefore it is difficult at the moment to establish if extensive enhancement is just a surrogate marker for molecular subtypes in non-WNT/SHH tumours because this requires multivariate analysis in the same group of patients. In our series, extensive enhancement was slightly more predictive than LCA pathology but number of Group 3 and 4 tumours was too small for such analyses. Further investigation should elucidate the relationship between already established prognostic features and the enhancement pattern.

Nevertheless, it is important to underline that our survival analyses were performed within the biologically relevant group of non-WNT/SHH tumours since WNT and SHH tumours have distinctive characteristics and should be analysed separately as independent cohorts. Very recent results published by Hervey-Jumper et al. [19] indicated that the presence of enhancement did not correlate with worse patient prognosis, but the results were not related to biological subtypes. It was noticed in our work that majority of SHH tumours were extensively enhancing but patients are long term survivors. Therefore inclusion of patients with SHH tumours to overall population may have an impact on final results when testing enhancement pattern.

In summary, we propose that hemispheric cerebellar location can serve as a surrogate marker for SHH medulloblastoma. In spite of this, laboratory tests should be introduced to identify WNT and SHH tumours because, for example, not all SHH tumours are hemispheric. The remaining non-WNT/SHH tumours can be classified according to the gadolinium enhancement pattern into two prognostic categories. Independent studies are necessary to validate and extend our findings, and provide a biological explanation for identified correlations.

## Electronic supplementary material

Supplementary material 1 (PDF 30 kb)

Supplementary material 2 (XLS 46 kb)
