# Supplementary material for: Contrast enhancement pattern predicts poor survival for patients with non-WNT/SHH medulloblastoma tumours
Source: J Neurooncol. 2015 Apr 11;123(1):65–73. doi: 10.1007/s11060-015-1779-0 (PMC4439433; doi:10.1007/s11060-015-1779-0)
Supplement: Supplementary file 1 — Supplementary material 1 (PDF 30 kb) [file 11060_2015_1779_MOESM1_ESM.pdf]

POLISH PAEDIATRIC NEUROONCOLOGY GROUP (PPNG)

MEDULLOBLASTOMA IN CHILDREN OLDER THAN 3 years TREATMENT PROTOCOL

1. STANDARD RISK GROUP (non-anaplastic tumor, totally or subtotally resected (tumor rest less than 1,5cm3), no tumor cells in the csf, no visible metastases in the initial MRI of the brain and spine)

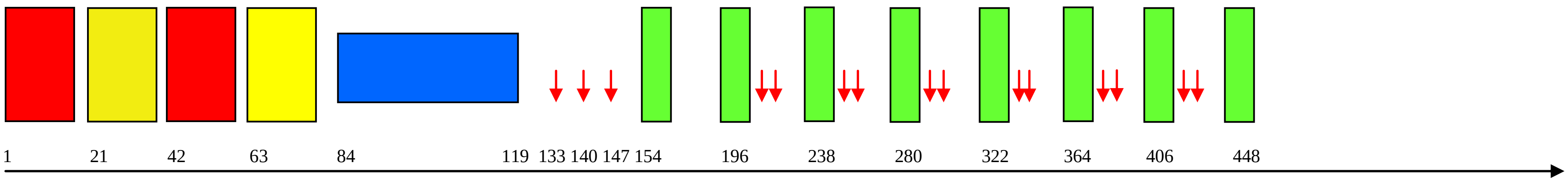

Vincristine 1,5 mg/m2 d1(d42), Etoposide 100mg/m2 d 1,2,3 (d42,43,44), Carboplatin 500 mg/m2 d 1,2(d42,43)

Vincristine 1,5 mg/m2 d21(d63), Etoposide 100 mg/m2 d 21,22,23 (d63,64,65), Endoksane 1500 mg/m2 d 21, (d63)

Radiotherapy 25 Gy on the axis, 55 Gy to the tumor bed

Vincristine 1,5 mg/m2 d 133, 140, 147

Vincristine 1,5 mg/m2, Cisplatin 75mg/m2, Lomustin 75mg/m2 d154,196,238,280,322,364,406,448,

2. HIGH RISK GROUP (LCA medulloblastoma or tumor rest > 1,5 cm3 or tumor cells in the csf (+), or any visible metastases in the MRI of the brain or spine)

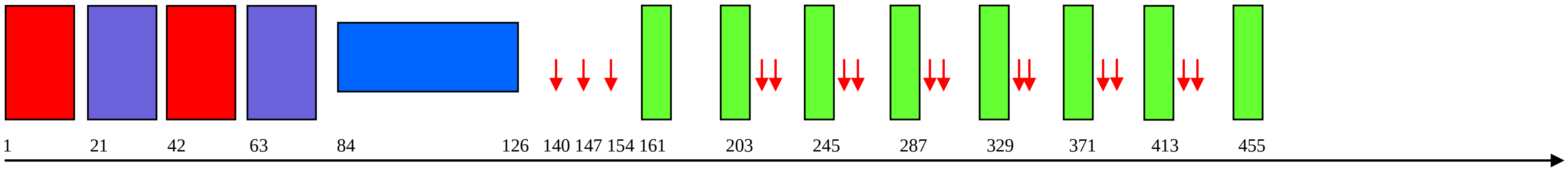

Vincristine 1,5 mg/m2 d1(d42), Etoposide 100mg/m2 d 1,2,3 (d42,43,44), Carboplatin 500 mg/m2 d 1,2(d42,43)

Etoposide 60 mg/m2, Ifosfamide 900 mg/m2, Cisplatin 20 mg/m2 d 21,22,23,24,25 (d63,64,65,66,67)

Radiotherapy 36 Gy on the axis, 55 Gy to the tumor bed, boost on the metastases that didn't regress after chemotherapy

Vincristine 1,5 mg/m2 d 140,147,154

Vincristine 1,5 mg/m2, Cisplatin 75mg/m2, Lomustin 75mg/m2 d 161, 203,245,287,329,371,413,455
